# HIV self-test reporting using mHealth platforms: A pilot study in Johannesburg, South Africa

**DOI:** 10.3389/frph.2023.1073492

**Published:** 2023-02-27

**Authors:** Mothepane Phatsoane Gaven, Matthew Quaife, Mohammed Majam, Leanne Singh, Naleni Rhagnath, Theodore Wonderlik, Siphamandla Bonga Gumede

**Affiliations:** ^1^ Ezintsha, Faculty of Health Sciences, University of the Witwatersrand, Johannesburg, South Africa; ^2^ Department of Global Health and Development, London School of Hygiene & Tropical Medicine, London, United Kingdom; ^3^ Department of Interdisciplinary Social Science, Utrecht University, Utrecht, Netherlands

**Keywords:** HIV testing services, HIV self-testing, linkage, HIV care, monitoring and evaluation, short message services, IVRS

## Abstract

**Background:**

The main impediment to operational scale-up of HIV self-testing (HIVST) and counselling, is a dearth of information on utilisation, reporting, and linkage to care for HIV-positive individuals. To inform solutions to this issue, this study investigated the utility of self-testers reporting their results using a mobile-health (mHealth) platform, and whether seropositive users linked into care.

**Method:**

Candidates who met the recruitment criteria across multiple sites within inner-city Johannesburg each received an HIVST kit. Using short message service (SMS) reminders (50% standard and 50% behavioural science), participants were prompted to self-report results on provided platforms. On the seventh day, users who did not make contact, were called, and surveyed *via* an interactive voice response system (IVRS). Multivariable regression was used in reporting by age and sex.

**Results:**

Of the 9,505 participants, 2,467 (25.9%) participants answered any survey question, and of those, 1,933 (78.4%) were willing to self-report their HIV status. Men were more likely than women to make an inbound call (10.2% vs. 9.1%, *p* = 0.06) however, women were significantly more likely to self-report their test result (AOR = 1.12, 95%CI = 1.01–1.24, *p* = 0.025). Overall, self-reporting a test result was predicted by being younger and female. In addition, reporting HIV results was associated with age, 25–35 (AOR = 1.58, 95% CI = 1.24–2.02) and above 35 years (AOR = 2.12, 95% CI = 1.61–2.80). Out of 1,933 participants willing to report their HIV status, 314 reported a positive test, indicating a HIV prevalence of 16.2% (95% CI: 14.6%–18.0%) and of those 204 (65.0%) reported inclination to link to care.

**Conclusion:**

While self-reporting HIVST results *via* an IVRS system yielded a higher response rate, behavioural SMSs were ineffective in increasing self-reporting.

## 1. Introduction

In 2019, South Africa had an estimated 7.5 million people living with HIV (PLHIV), approximately 6.9 million (92%) of whom knew their status. The country faces the highest HIV burden and implements the largest HIV treatment programme globally, yet despite the steady progress towards achieving the UNAIDS 90–90–90 targets, achieving the second 90 (all people diagnosed with HIV will be allowed to start ART treatment) remains problematic. In South Africa, only 5.2 million (70%) of HIV positive people are receiving HIV treatment (1). Further, estimates indicate that there is an HIV incidence (per 1,000 population) of 6.9 in the young adult population between the ages of 15–49 (2, 3). Testing is not being taken up equally, with men in general and adolescent girls and young women, being harder to reach with traditional HIV testing modalities. South Africa employs a multi-pronged HIV prevention approach (3). Despite South Africans being able to access the cutting edge of biomedical prevention and testing services, new infections remain high, and low uptake and coverage of existing HIV testing services pose a significant challenge for universal access to HIV treatment (4). In March 2018, the South African National Department of Health approved and issued guidance on the use of HIV self-tests (HIVSTs) (5).

According to the World Health Organization (WHO), HIVST is defined as the process whereby an individual collects their own specimen (blood or oral fluid), performs HIV testing using an HIV rapid diagnostic test and interprets the result themselves either assisted or unassisted (6). HIVST is not intended to replace facility-based HIV tests but rather serves as the initial step to knowing one's HIV status, and where seropositivity needs a further confirmatory test in the health facility. In July 2017, the OraQuick HIV 1/2 test was approved by WHO as the first pre-qualified HIVST, and in January 2019, the INSTI HIV ½ HIVST was approved (7). Yet, despite the approval of HIVSTs for use in South Africa since 2018, the utilisation rate is (85%), and HIVST users do not utilise the health system fully to obtain a confirmatory test and link to care after receiving a positive HIVST test (8). Linkage to care (prevention services or treatment) is one of the most important aspects of non-facility-based testing. Globally, there is a shortage of available data that demonstrates effective linkage to care for individuals that have self-tested. A systematic review by the WHO has reported on only two randomised controlled trials (RCTs) having examined linkage to care (8). One RCT found that 72% of the male partners of women who received an HIVST kit accessed further testing to confirm their result, even though this could not be directly compared with standard testing. Johnson C, et al. reported lower linkage to care than those diagnosed in the standard group, which was partly attributed to few HIV-positive test results, under-reporting, and possibility that some men knew their HIV-positive status already (8).

Like HIVSTs, mHealth technologies could potentially improve access to public health efforts in underserved communities by making healthcare services easier and more convenient to access (9). mHealth tools can comprise online videos, apps and text messages, depending on the technology available to users. Reviews of interventions implemented globally report strong evidence of text/short message service (SMS) intervention effectiveness for diabetes self-management, weight loss, physical activity, and smoking cessation (10). Within HIV studies, there is strong evidence that SMS messages increase the propensity for HIV positive persons to adhere to treatment (11, 12). Recent studies conducted across the income spectrum report high antiretroviral therapy adherence with the use of mobile phones (standard or smartphone) through interactive voice response calls, and SMS (13). Such phone-based systems offer a low cost and consistent delivery of messages. Patients use the keypad or voice response to choose menu options, respond promptly and answer questions (14).

There is currently no evidence for low- and middle-income countries on whether SMS messages increase linkage to care after an HIV self-test. Evidence from the UK suggests that mHealth interventions to increase HIVST reporting can be enhanced through the inclusion of behavioural insights or “nudges”, where small changes in wording led to increased reporting (15). However, there is no evidence on the effectiveness of whether messages based on behavioural nudges can affect HIVST reporting in the South African context. Our study fills these gaps by reporting data from a pilot study of an mHealth communication platform which used SMS and interactive voice response system (IVRS) to enhance HIVST reporting and, if positive, linkage to care. Our primary outcomes report utilization of the healthcare system, reporting of HIV status, and whether these outcomes varied across participant demographics, or whether they received SMS messages based on behavioural nudges.

## 2. Methods and design

### 2.1. Setting and recruitment

The study was multi-site and aimed to recruit 12,000 participants from 35 sites in and around the city of Johannesburg. Recruitment sites were heterogenous, and were all densely populated, and included malls, shopping centres, colleges, taxi ranks and informal housing settlements. The fieldwork team consisted of four staff, who presented at each site with a branded canopy, materials advertising HIVST distribution, test kits, and data collection tools. All willing participants were individually approached and provided with information regarding the study. If the participant was interested and met al.l the inclusion criteria, they were consented after which their details were collected using paper-based data collection tools and captured onsite on the Viamo platform. Participants were also provided with an HIVST kit together with information leaflet to contact a hotline or access a website link to report their HIVST result. All staff were comprehensively trained on the study protocols, including requesting informed consent, referral of self-reported HIV positive participants, and how to use the study database.

### 2.2. Mhealth intervention

On enrolment, participant details were entered into the mHealth system of Viamo Mobile, and participants encouraged to conduct a short survey *via* recorded phone line or website to self-report test use and result. The system encouraged self-reporting through two SMS messages sent at three- and five-days post registration. On the seventh day after distribution, if the participant had not initiated contact with the system, an interactive voice response system (IVRS) called the participant to go through a short survey which included test result. The recorded voice menu options were identical irrespective of whether respondents called in or were called by the system. First, participants were asked if they had used the test and if they were willing to reveal their result. Then, participants were asked how easy the test was to use, how much they would be willing to pay for the test if it had not been provided for free, when they last tested for HIV, when they planned to test next for HIV, and their willingness to pay for the test if it had not been provided free of charge.

### 2.3. Inclusion and exclusion criteria

Participants were eligible for inclusion if they showed understanding of the written informed consent process, were aged 18 years and older, had access to a cellular phone able to receive SMS messages, and were able to speak and read English and had not tested for HIV in the 3 months prior. Study tools and the consent forms were in English which is a commonly used language in the recruitment areas. Participants were excluded from the study if they were not able and willing to provide informed consent, could not provide a verifiable mobile phone number, had tested in for HIV in the last 3 months and had any condition which would render them unsuitable or unsafe for enrolment, for example pregnancy, being intoxicated or having an acute illness. Participants were not excluded based on race, gender, ethnicity, or sexual orientation.

### 2.4. Informed consent, enrolment and data collection

Upon enrolment, participants were asked to report their age and gender – no other data were collected to make data collection light-touch and reflect real-world conditions as much as possible. Study staff explained the material included in the screening kit, which included written information on how to report a result. The kit also included a linkage referral card for participants wishing to report to one of a list of named referral clinics in the area for confirmatory testing. All enrolled participants consented to be contacted by the study *via* phone or SMS at a later date. Participant data were collected on paper, and immediately entered into the study database using a computer tablet. Digital data were securely stored on Viamo Mobile servers and reviewed daily by the study team and supervisors for errors. Study tools and consent forms were stored in an access-controlled data room.

### 2.5. Behavioural wording in SMS messages

**Figure 1:**
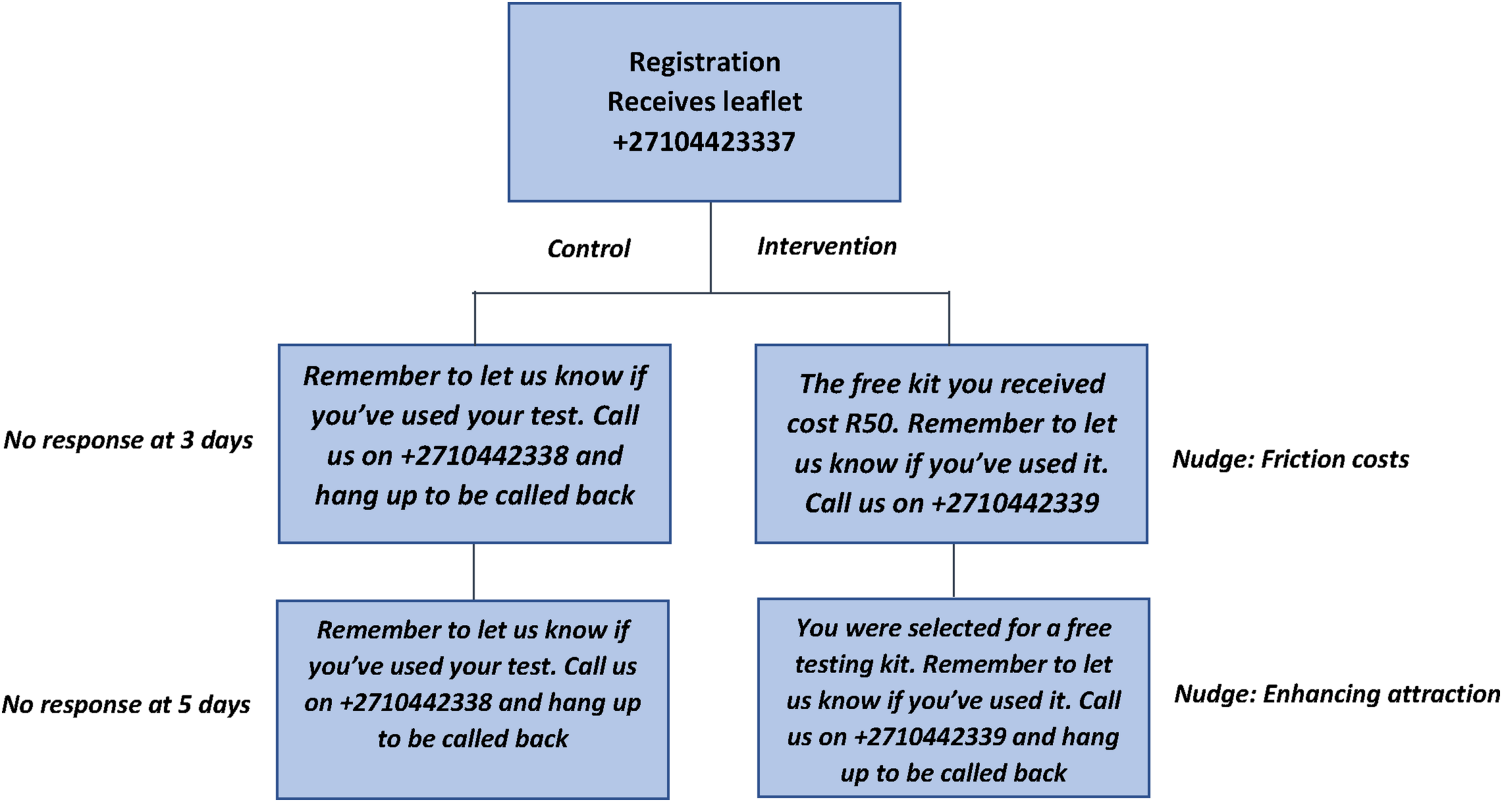
Workflow design of behavioural SMS received by participants.

Since other HIVST programmes have shown that incorporating SMS messaging increased the reporting of test use and results (16), we deemed it appropriate for participants to receive any SMS message prompts. Evidence from the UK suggested that SMS messages based on behavioural nudges could be effective in increasing HIVST response rates. We adapted this intervention based on the Easy, Attractive, Social and Timely (EAST) framework (17), through discussion with researchers and HIVST experts. Upon enrolment participants were randomly allocated to receive one of two sets of test messages, as shown in Figure 1. Participants in the control arm received the same prompt on days three and five. On day three, participants in the intervention arm who had not yet responded, received a message highlighting the cost of the test they received, based on the principle of highlighting friction costs to participants (17, 18). On day five, participants who had not yet responded received a message seeking to enhance the attractiveness of the HIVST. Outcomes were pre-specified in a submission to the American Economic Association Trial Registry (AEARCTR-0002409) as a report of HIVST use through contacting the mHealth system, and HIVST result.

### 2.6. Data analysis and outcome measurement

Data were downloaded from the Viamo Mobile system to Stata 15 (StataCorp; College Station, United States) where they were reshaped and analysed. We used participant's phone numbers as an identification tool. Based on our experience, we concluded that asking participants to report the number written on their test kit would be ineffective in tracking them through the system, due to a possibility of having duplicate phone numbers in the system, as participants collected more than one kit. We assessed whether duplicates were genuine, and not artefacts such as data entry errors, by looking for different test kit IDs and noting if the place, time, and date of participation was substantively different. Our main analysis omits duplicates and only considers the first time a phone number entered the system, though we conducted sensitivity analyses estimating results using the full dataset and explored reporting by duplicate phone numbers. We use multivariable regression to explore variation in reporting by age and gender, the only participant level characteristics collected.

Outcome measures included the proportion of people who engaged with the mHealth system at different times through SMS messages and voice calls, the proportion of people willing to report their HIV status, and associations with age and gender.

### 2.7. Ethical review

The study and related study material was reviewed and approved by the Human Research Ethics Committee (HREC) of the University of the Witwatersrand (approval number 171113) and the Observational/Interventions Research Ethics Committee of the London School of Hygiene and Tropical Medicine (reference 14485). Participants were not reimbursed.

## 3. Results

In total 10,698 participants registered a phone number with the system and received an HIVST kit. Of those, 9,504 (89%) were unique phone numbers; this figure is used as the denominator for the main analysis. 183 participants (1.9%) called in before being contacted by SMS on day 3. There were slightly more male (5,047, 53%) than female (4,457, 47%) registrations (Table 1). Almost half of the participants (4,501, 47%) were between 25 and 35 years old (Table 1).

**Table 1:** Demographics.

Participant Demographics (*n* = 9,505)			
Characteristic		Frequency (*n*)	Percentage (%)
Age group	18–24	2,497	26.27%
	25–35	4,501	47.36%
	36+	2,503	26.34%
	Not answered	3	0.03%
Sex	Female	4,457	46.90%
	Male	5,047	53.10%
	Not answered	0	0.00%

### 3.1. Description of participant interactions with the mHealth system

**Figure 2:**
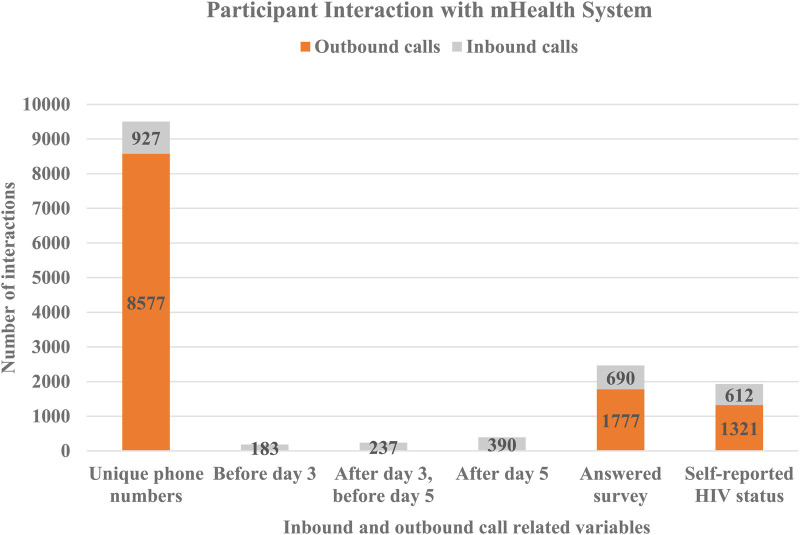
Participant survey response.

On day three and day five after registration, participants received an SMS message prompting them to call into the system and complete the survey. This increased self-reporting five-fold compared to unprompted responses before day three (Table 2). Two hundred and thirty seven (237, 2.5%) participants of those who had not called before day three) participants called and completed the survey after receiving the SMS message on day three, and before receiving the second reminder on day five (Figure 2). One hundred and twenty three (123, 1.3%) of those who had not called before day five) called after receiving a second reminder on day five. In total, 1,933 (20.3% of total participants) reported results within seven days (both inbound and outbound), 690 (35.7%) of whom answered any survey question and 612 (31.7%) self-reported their HIV status (Figure 2). Men were slightly more likely than women to have made an inbound call (10.2% vs. 9.1%, *p* = 0.06) but significantly less likely to have reported a test result (19% vs. 22%, *p* = 0.01).

**Table 2:** Self reporting call status (inbound and outbound indicators) for the study participants disaggregated by sex.

Self-reporting Call Status			
Indicators/variables	Sex	Number (*n*)	Percent (%)
Unique numbers	Male	5,047	53.10
	Female	4,457	46.90
Inbound before day 3 (*n* = 183)	Male	93	50.82
	Female	90	49.18
Inbound day 3 (*n* = 237)	Male	112	47.26
	Female	125	52.74
Outbound day 3 (*n* = 4,140)	Male	2,007	48.48
	Female	2,133	51.52
Inbound day 5 (*n* = 123)	Male	72	58.54
	Female	51	41.46
Outbound day 5 (*n* = 4,136)	Male	2,013	48.67
	Female	2,123	51.33
Outbound day 7 (*n* = 8,109)	Male	4,358	53.74
	Female	3,751	46.26
Inbound and outbound self-reporting (SR) (*n* = 1,933)	Male	976	50.49
	Female	957	49.51
SR HIV results (*n* = 612)	Male	320	52.29
	Female	292	47.71
SR HIV positive results (*n* = 314)	Male	165	52.55
	Female	149	47.45

The remaining 8,109 (85.3% of total) of participants received a phone call seven days after enrolment by the same recorded phone survey as accessed by those calling into the system (Table 2). Of these, 1,777 (20.7% of those called) answered the first question of the survey and 1,321 (15.4% of those called) self-reported HIV status. Compared to unprompted and prompted SMS messages, the call out led to 1.9 times as many responses and 2.2 times as many self-reports of HIV status.

### 3.2. Self-reporting of HIV status

In total, 2,467 (26.0%) participants answered any survey question, and 1,933 (78.4%) of those (20.3% of total) were willing to self-report their HIV status. Women were significantly more likely to self-report their test result (AOR = 1.12, 95%CI = 1.01–1.24, *p* = 0.025). Self-reporting a test result was predicted by being younger and female, though these were not significant predictors of a positive HIVST result. In addition, reporting HIV results increased incrementally with age, 25–35 (AOR = 1.58, 95% CI = 1.24–2.02) and above 35 years (AOR = 2.12, 95% CI = 1.61–2.80). Out of 1,933 self-reporting participants, 314 reported a positive test, indicating a HIV prevalence of 16.2% (95% CI: 14.6%-18.0%). There was indicative evidence (*p* = 0.426) that HIV prevalence was slightly higher among men (16.9%, 95% CI: 14.6–19.4%), than women (15.6%, 95%CI: 13.3–18.0%), although Table 3 shows that neither sex nor age was predictive of reporting an HIV positive test result (Table 3).

**Table 3:** Logistic regression results for self-reporting status and reporting HIV positive result.

Logistic regression results for self-reporting status and reporting HIV positive result												
Variable	Self-reported results (inbound and outbound)				Inbound self-reporting results				Self-reporting HIV positive results (inbound and outbound			
	Unadjusted odds ratio (95% CI)	*p*-value	Adjusted odds ratio (95% CI)	*p*-value	Unadjusted odds ratio (95% CI)	*p*-value	Adjusted odds ratio (95% CI)	*p*-value	Unadjusted odds ratio (95% CI)	*p*-value	Adjusted odds ratio (95% CI)	*p*-value
**Age**												
25–35 years (vs. 18–24)	0.95 (0.84–1.07)	0.378	0.96 (0.85–1.08)	0.480	1.59 (1.25–2.02)	**<0** **.** **001**	1.58 (1.24–2.02)	**<0** **.** **001**	0.98 (0.73–1.30)	0.882	0.97 (0.73–1.29)	0.823
Above 35 years (vs. 18–24)	0.81 (0.71–0.94)	**0** **.** **004**	0.83 (0.72–0.95)	**0** **.** **009**	2.14 (1.63–2.81)	**<0** **.** **001**	2.12 (1.61–2.80)	**<0** **.** **001**	0.97 (0.70–1.36)	0.878	0.95 (0.68–1.34)	0.784
**Gender**												
Female (vs. Male)	1.14 (1.03–1.26)	**0** **.** **010**	1.12 (1.01–1.24)	**0** **.** **025**	0.90 (0.74–1.09)	0.282	0.97 (0.80–1.18)	0.772	0.91 (0.71–1.15)	0.426	0.90 (0.71–1.15)	0.413

### 3.3. New diagnoses and linkage to care

Out of 314 respondents reporting an HIV positive test, 130 (41.4%) (70 males and 60 females) reported that this was the first positive HIVST that they had taken. Also, of the 314 respondents reporting a HIV positive test, 204 (64.9%) (116 males and 88 females) reported that they had either linked to care or intended to link to care.

### 3.4. Ease of using the test

Of the 2,467 participants who answered any survey question, 1,592 (64.5%) reported that the HIVST was very easy or easy to use; women were significantly more likely to report this than men (*p* = 0.03).

### 3.5. Amount willing to pay

Half of the sample were asked to enter the amount willing to pay without prompts for amount categories – the mean amount willing to pay reported by these respondents was ZAR 70.39 (±4.77 USD) (95% CI: 64.3–76.4), and median ZAR 50 (±3.34 USD) (IQR: 10–100). Furthermore, among those who were presented with varying price point options to report the amount willing to pay, the pay category of ZAR 10–50 (±0.68 USD–3.34 USD), being the cheapest option was chosen by 71.0% of respondents. Then 17.2% of respondents were willing to pay ZAR 50–100 (±3.34 USD–6.78 USD), 6.0% ZAR 100–150 (6.78 USD–10.16 USD) and 6.0% ZAR 150+.

### 3.6. Previous testing behaviour and intention to test again

As shown in Table 4, a relatively high proportion of respondents had tested in the previous 6 months (74.4%), and after using a HIVST the intention to test again in the next three months was also high (77.0%).

**Table 4:** Previous testing behaviour and intention to test again.

Testing Behaviour					
Time of last test (*n* = 1,697)	*N*	%	Anticipated time to next test (*n* = 1,671)	*N*	%
Last 3 months	1,001	59.0%	In 3 months	1,287	77.0%
Last 6 months	261	15.4%	In 6 months	202	12.1%
Last year	203	12.0%	In 1 year	75	4.5%
Last 2 years	61	3.6%	In 2 years	20	1.2%
Don't know	171	10.1%	Don't know	87	5.2%

### 3.7. Impact of behavioural SMS wording on responses

There was no evidence of differences in age or gender among participants randomised to receive standard or behavioural SMS messages and of the 9,505 unique phone numbers, 4,637 (48.8%) received behavioural SMS. Overall, the impact of behavioural SMS messages was small, but there was very weak evidence that behavioural SMS led to lower engagement with the mHealth system. Those who received behavioural SMS messages were slightly less likely to answer any survey question (1,175, 25.3%) compared to the control arm (1,291, 27.8%), indicating a difference of −1.2 percentage points (95% CI: 0.5–2.9%, *p* = 0.2). There was no difference in the likelihood of reporting a positive result between intervention and control arms (*p* = 0.44). A secondary analysis, which was not pre-specified and should therefore not be interpreted as causal, found that those who received behavioural SMS messages were slightly less likely to make an inbound call (424, 9.1%) compared to the control arm (503, 10.8%) with a difference of −1.2 percentage points (95% CI: 0–2.4%, *p* = 0.05).

## 4. Discussion

The study purpose was to determine if mobile health communication platforms (SMS and voice calls using behavioural science principles would have an impact on self-testers reporting their results and contributing to increased linkage to care.

Overall results showed that 9.8% shared results within 7 days after up to two SMS prompts, and 20.7% of remaining non-responders shared results when they received an automated outbound call. Out of the 314 HIV positive respondents, 65% reported to have had either linked/intend to link to care. Willcox JC, et al. suggest that the effectiveness of text-messaging can be enhanced when coupled with the use of other mediation strategies like links to YouTube videos and webpages (19). Given that in our study participants' face-to-face engagement with the study team ended once their details were recorded and they had received their HIVST, it is possible that some participants did not link to care because they did not completely. For future similar research, it may be advantageous to test whether responses increase if participants could access for example, a video which reiterates the explanations and guidance which they were given on recruitment. Noteworthy too, is that the wording of the SMS message had no impact on response rates. While data on the literacy of the participants and type of language preference were not collected, the impact of using English as the sole medium of communication for this study needs to be considered. In Venables E, et al.'s qualitative study exploring the effectiveness of using SMS communication in viral load mitigation, some patients revealed that receiving messages in English (as opposed to a local language) was problematic (20). Similarly, literacy levels of our study participants needed to be taken into account, since apart from English, participants' first language could well be any one of South Africa's other ten official languages. A study by Sineke T, et al. which focused on HIV knowledge among HIV positive patients in Johannesburg, concluded that HIV knowledge corresponds with English literacy levels (21).

The results indicate that there is a fair degree of acceptability in engaging with text message prompts to report HIV results as well as the intention to link to care. The value of using an mHealth platform like text messages is exemplified in a Ugandan study which found that the high acceptability and feasibility of using mHealth to track HIV positive youth who were lost to follow-up, resulted in the Ministry of Health endorsing mHealth as a strategy to support HIV care (22). Even though the participants of our study did demonstrate health-seeking behaviour, a number of them in fact either did not engage with the text messaging at all or partially engaged. One potential reason for this is that participants were probably sceptical about responding to the contact numbers used for prompting response. This is because the Viamo platform numbers used to prompt the participants were unfamiliar compared to locally used South African numbers. In addition, participants were unsure whether engaging in text messaging would come with incurred costs. Therefore, participants' hesitancy to use the provided numbers could have negatively impacted participants' willingness to respond.

Our study reported a larger number of participants who had tested positive for HIV responding to calls after seven days, compared to the number of those who tested positive calling in before seven days. This limited and delayed response of HIV positive testers using the mHealth services is similar to the findings in another study of HIV positive patients' behaviour on receiving SMS reminders to report to clinics for information about their viral loads (20). It was concluded that while SMS had the potential to reach large numbers of people quickly, it did not significantly affect the turnaround time for patients reporting to clinics. Furthermore, a concern that was raised in that study was that a reminder message that lacked detailed explanations could cause undue stress to the recipients, negatively impacting on their response time (20). This concern is significant in that it could point to a reason for the post-HIVST reaction time of participants in our investigation. It is possible that increased levels of stress and fear caused by HIV-positive readings and the perceived repercussions thereof, caused delays in reporting results. Other possible barriers to using the mHealth services, could be that in addition to being wary of the costs associated with using their mobile phones, participants also and/or had limited understanding of the expectations post-HIVST. These possibilities were corroborated in the conclusions of a recent similar South African study (23).

While the behavioural SMS messages intended to nudge engagement with the mHealth platform, data showed that they had no impact in doing so, indicating the opposite impact of behavioural messages on reporting that was recorded in a UK study (14). The limited effect documented in our study suggests that more simplistic and direct messaging could potentially yield better engagement in the South African setting. Also, the relatively high response rates from SMS prompts, regardless of the wording, indicate that the impact of sending any SMS message may be much greater than the wording of such messages. A study by Bidargaddi N, et al., found that the context of participants influenced when they were most responsive to prompts – for example, in some contexts, on weekends responsiveness increased within 24 h when prompts were sent around lunch time (24). Given that in South Africa such a range of contexts exists amongst its people, more studies should be conducted to assess the extent to which patterns related to time emerge (if at all) regarding both inbound and outbound responses across different groups, the results of which can inform further similar impact studies.

Unfortunately, technical issues with the mHealth system were experienced during the study such as the system delaying in sending messages in real time once the participant had been registered, therefore affecting user interaction with the platform. Amongst the other reasons, the system used different numbers to prompt the participants response therefore leading to reluctance in answering the call. Apart from potentially willing participants being unable to participate in the study as intended, technical difficulties have wider implications. Greve M, et al., explain that too few pilot mHealth projects in low-resource environments are able to progress to the sustainability stage, and this then hampers the development of healthcare interventions, essentially placing universal health coverage at risk (25). The aforementioned technical complications have also been identified by Mbunge E, et Al. in their review of the utilisation of mHealth in South Africa during the COVID-19 pandemic, and they encourage the revision of mHealth policies as well as political and fiscal investments into the sustainability of mHealth (26).

In their investigation of the design and value of mHealth platforms for HIV care, Marent B, et al. suggest that these platforms are most effectively used by people who already were aware of their HIV positive status (27). If this is indeed the case, further investigation needs to be conducted into why this is so in comparison to people who are unaware of their HIV status. The results thereof ought to be used to inform the development of mHealth programmes that are more feasible for sustained use by first-time testers.

There is clear evidence that SMS reminders and outgoing phone calls were acceptable and resulted in relatively high levels of HIVST result self-reporting. This study adds to the evidence that mHealth systems may have an important role in engaging and communicating with health system users. Nonetheless, an important concern is the financial implications for users of HIVST and mHealth services. Participants who did not receive prompts were willing to pay around ZAR 50–70 for the HIVST, whereas the large majority of those who were prompted selected the cheapest option – ZAR 10–50. This suggests that the cost of HIVST kits, if higher than ZAR 50 could be a deterrent to future uptake of HIVST in South Africa. Furthermore, one cannot out-rule the possibility that some participated in the study because they were given an HIVST for free and may in fact not be able to pay for other HIVST kits. This implies that governmental subsidies may be necessary to scale up and ensure high HIVST uptake.

Healthcare communication through mHealth platforms is increasing, with trends exacerbated by health system changes during the COVID-19 pandemic. This study evaluated a platform using a combination of short message service and voice call prompts to initiate HIV self-reporting and linkage to care around HIVST. For HIV self-reporting and linkage to care to be widespread and efficient, electronic health, also known as eHealth methods, can play a key role in the health system moving forward. Given the ubiquity of mobile phones, Mechael PN, as early as 2009, identified their value to mHealth in boosting communication and access to information (28). Lupton D, takes the idea of mHealth even further, in illustrating how with the inclusion of contemporary digital technology, mhealth has the ability to benefit not only patients, but societies and governments too (29). However, while the opportunities to exploit the mHealth, possibilities in providing healthcare are seemingly endless. There still remains questions around what conditions encourage and create the optimum usage of mHealth programmes and applications, in the self-reporting of HIV status and ensuing healthcare.

### 4.1. Strengths and weaknesses of the study

One strength of the study was that it targeted young men and women roughly equal in number and recruited a large number of participants using street-based recruitment in urban Gauteng Province; previous studies have struggled to engage men in HIVST distribution and results reporting (30). According to Chikovore's findings, men preferred traditional medicine and also that primary health care settings were not welcoming for them (reference).

A limitation of this study is that the system did not permit sharing of phone as the phone number of the participant was used as a unique identifier. Thus, there is a need to explore more improved mHealth systems that have a multiple-user function that caters for various user identities to engage with the system.

Using English as the sole medium of communication for this study posed a serious limitation as some patients revealed that receiving messages in English was problematic.

This study included a small number of people who responded to the questions and was limited to one study region (Gauteng, South Africa). Therefore, findings from this study may not be generalizable to other settings in South Africa, or to other country settings or larger populations.

## 5. Conclusion

HIVST empowers individuals to test and know their HIV status privately, safely, and easily. It has been driven to complement, not replace traditional HIV testing services to reach populations that are otherwise not testing. This study demonstrated that HIVST is feasible and acceptable to the target populations with uptake and acceptance of tests being very high.

Reporting results from HIVST distribution programmes remains one of the main barriers to wide-scale implementation and acceptance of this testing modality. Implementers need to weigh up the benefit of HIVST more easily reaching the target population against the downside of not having the patients actionable result immediately available. While self-reporting of results being inherently biased, is not a true reflection of the actual incidence rate, there still is value to be derived from this approach as it provides estimates of incidence and linkage into care.

Self-reporting HIVST results *via* an IVRS system positively impacted the response rate. The automated nature of the IVR system allowed for a consistent follow up across all tests distributed, irrespective of whether the survey was responded to. Patients drop off on IVRS calls as it progressed showed patient reluctance to spend more than a minute following prompts and answering survey questions, and this needs to be taken into consideration in the further development of this self-reporting mechanism. Furthermore, since behavioural SMSs were ineffective in increasing self-reporting, other factors like timing of calls need to be considered.

Overall, IVRS reporting of results in self-testing programmes is not ideal for HIVST programmes to overcome reporting problems. It should be offered as part of a bouquet of options to patients/clients to allow the end user to engage with a reporting tool of their choice, if at all. Further studies are needed to evaluate which self-reporting tools have the highest impact and effectiveness.

## Data Availability

The raw data supporting the conclusions of this article will be made available by the authors, without undue reservation.
